# Development of a Nanostructured Electrochemical Genosensor for the Detection of the K-ras Gene

**DOI:** 10.1155/2022/6575140

**Published:** 2022-10-17

**Authors:** Luis Fernando Garcia-Melo, Miguel Morales-Rodríguez, Eduardo Madrigal-Bujaidar, Eduardo O. Madrigal-Santillán, José Antonio Morales-González, Rosa Natali Pineda Cruces, Jorge Alfredo Campoy Ramírez, Pablo Damian-Matsumura, Alexandro Tellez-Plancarte, Nikola Batina, Isela Álvarez-González

**Affiliations:** ^1^Division de Ingeniería en Nanotecnología, Universidad Politécnica del Valle de México, Av. Mexiquense s/n esquina Av. Universidad Politécnica, Tultitlan Estado de México, CP 54910, Mexico; ^2^Laboratorio de Nanotecnología e Ingeniería Molecular Área Electroquímica, Departamento de Química, CBI, Universidad Autónoma Metropolitana-Iztapalapa (UAM-I), Av. San Rafael Atlixco 186, Iztapalapa, CP 09340, México City, Mexico; ^3^Laboratorio de Genética, Escuela Nacional de Ciencias Biológicas, Instituto Politécnico Nacional, Avenida Wilfrido Massieu s/n Col. Zacatenco Del. Gustavo A. Madero, CP 07738, Ciudad de México, Mexico; ^4^Laboratorio de Medicina de Conservación, Escuela Superior de Medicina, Instituto Politécnico Nacional, Unidad Casco de Santo Tomás, Plan de San Luis y Díaz Mirón, Ciudad de México, CP 11340, Mexico; ^5^Laboratorio de Endocrinología Molecular, Departamento de Biología de la Reproducción, CBS, Universidad Autónoma Metropolitana-Iztapalapa (UAM-I), México City, Mexico

## Abstract

In the scientific literature, it has been documented that electrochemical genosensors are novel analytical tools with proven clinical diagnostic potential for the identification of carcinogenic processes due to genetic and epigenetic alterations, as well as infectious diseases due to viruses or bacteria. In the present work, we describe the construction of an electrochemical genosensor for the identification of the k12p.1 mutation; it was based on use of Screen-Printed Gold Electrode (SPGE), Cyclic Voltammetry (CV), and Atomic Force Microscopy (AFM), for the monitoring the electron transfer trough the functionalized nanostructured surface and corresponding morphological changes. The sensitivity of the genosensor showed a linear response for the identification of the k12p.1 mutation of the *K-ras* gene in the concentration range of 10 fM to 1 *μ*M with a detection limit of 7.96 fM in the presence of doxorubicin (Dox) as DNA intercalating agent and indicator of the hybridization reaction. Thus, the electrochemical genosensor developed could be useful for the identification of diseases related with the *K-ras* oncogene.

## 1. Introduction

Cancer represents a health problem worldwide; in 2020, there were 19.3 million new cases and 10 million deaths due to this disease [1, 2]. In this same year, the cancers diagnosed with greatest frequency in men were those of lung, colon, stomach, and liver, while those for women were breast, colon and rectum, uterine cervix, and stomach. These data indicate that Colorectal Cancer (CRC) is the second most frequent tumor found in males and second in females, representing the third most common cause of death by cancer at the worldwide level [2]. It is noteworthy that a fundamental part of CRC initiation is related with genotoxic and epigenetic agents, which are capable of producing damage on the genetic material [3]. Genetic and epigenetic alterations have been observed in the colorectal carcinogenesis process; however, the mutation and loss of function of the K-ras oncogene is one of the most important, the most common mutations are p.G12D, p.G12V, and p.G13D [4, 5]. When *K-ras* gene is mutated, its GTPase activity is affected due to the lack of hydrolysis of active GTP and GAP-mediated guanine nucleotide exchange factors. In consequence, it is activated via the transduction pathway of growth signals and cellular differentiation, giving rise to the initiation, promotion, and/or progression of CRC [6, 7]. Some studies showed that the K-ras oncogene has the necessary characteristics to be used as a biomarker capable of preventing, diagnosing, and even predicting CRC [8–11]. Therefore, the precise identification of the mutations in K-ras oncogene nucleotides (DNA) can be of great relevance for the diagnosis of this type of disease or where this type of gene is expressed, such as, for example, in colon (40%), lung (30%), pancreas (80%), and thyroids (55%). [12].

Under this perspective, DNA represents one of the most important tools due to its multiple applications in medical investigation and clinical diagnosis [13]. At present, the detection and quantification of DNA is commonly conducted by Polymerase Chain Reaction (PCR) and the products obtained are analyzed by gel electrophoresis, in addition to Western and Southern blots [14–16]. Other methods utilized are PCR in real-time (qPCR) and digital PCR (dPCR), which perform amplification and detection simultaneously; these techniques are most employed during recent years for this type of analysis [15–17]. Unfortunately, the use of these types of equipment is limited to hospital laboratories or centers of investigation due to the complexity of the tests and the high cost of the reagents and materials. In this regard, there is a great challenge to achieve the rapid and effective analysis of DNA with minimal instrumentation at the patient level.

The biosensors purport to play a fundamental role in the identification of mutations and genetic disorders, which will allow the development of tools that provide detection and rapid diagnosis, being portable, low cost, and with high sensitivity and selectivity [18, 19]. The development of genosensors is based mainly on the immobilization of DNA probes on the working electrode surface and this process should possess a density and orientation of controlled packaging [20–23]. In this respect, the intercalating agents or electroactive molecules can interact with the DNA double strand, undergoing electrostatic interactions or intercalating themselves among the bases of genetic material, increasing the electrochemical response that arises from the association of the indicator with the DNA double strand, obtaining the hybridization signal as a result, and consequently improving the efficiency of the genosensors [23–26]. The sensitivity and selectivity of biosensors can be improved with nanostructures capable of preserving the activity of biomolecules. The common techniques to develop the nanostructures are layer-by-layer, Langmuir-Blodgett, and self-assembled monolayer (SAM) [27]. The latest system allows the nanostructuring of monolayers (SAMs) of alkylthiols at the genosensor surface to provide reproducible methodology, supplying control in terms of the desired orientation and distribution of the DNA sequences, with defined thickness, and three-dimensional structure. Moreover, such a system has the capacity to imitate biological membranes, which leads to wide biocompatibility [25–28]. The combination of these previously mentioned elements allows the creation of devices of nucleic acids and electrochemical transducers with high specificity and selectivity capable of analyzing samples of water, foods, viruses and other pathogens, soils, plants, or mutated genes; the monitoring of diseases, and as a tool for early diagnosis to prevent diseases like CCR [29, 30].

Based on the antecedents, the aim of the present research was to design and construct electrochemical biosensors of DNA with a high sensitivity capable of detecting the k12p.1 mutation of K-ras oncogene. To achieve this objective, we utilized Screen Printed Gold Electrode (SPGE), Cyclic Voltammetry (CV) as an electrochemical method, and Atomic Force Microscopy (AFM) to characterize the modification of the SPGE surface. Such devices can also be used for the study of other cancer related DNA mutations.

## 2. Experimental Study

### 2.1. Reagents

Ultrapure sodium dodecyl sulfate (SDS) was purchased from MP Biomedicals, Inc. (Solon, OH). Potassium ferrocyanide ([Fe(CN)_6_]^4−^) was obtained from Merck (Mexico). 2-mercaptoethanol (MER), 6-mercapto-1-hexanol (MCH), 11-mercaptoundecanoic acid (MUA), phosphate buffered saline pH 7.4 (PBS), ethylenediaminetetraacetic acid (EDTA), Trizma® hydrochloride (Tris-Cl), doxorubicin (Dox). Potassium ferricyanide ([Fe(CN)_6_]^3−^) was purchased from Sigma Chemicals (St Louis, MO). All solutions were prepared with ultrapure water from a Millipore Milli-Q system (resistivity 18.2 MΩcm) Millipore Corporation (Milford, MA). However, MUA was prepared with ethanol and ultrapure water.

Fully complementary target (K-ras) and its probe sequence, ssDNA, and sNC as non-complementary sequences were purchased from T4oligo Company (Irapuato, Gto). The oligonucleotide sequences used in the experiments were the following:

Probe Sequence (ssDNA): 5′-SH-(CH3)6- AAC TTG TGG TAG TTG GAG CT-3′

Fully complementary target (K-ras): 5′-AGC TCC AAC TAC CAC AAG TT-3′

Non-Complementary sequences (sNC): a) 5′-GAG GTT TGC AGA TCT CCA CC-3′.

All solutions were prepared in nuclease-free water. The stock solutions of oligonucleotides (DNA probe) were prepared in Tris-EDTA (10 mM:1 mM) buffer solution (pH 8.0) for a final concentration of 100 *μ*M. The stock solutions were kept frozen at −20°C. More diluted solutions of oligonucleotides were prepared in pH 7.0 PBS 0.01M. Electrochemical measurements were performed in redox probe solutions containing a drop of 150 *μ*L of 2.5 mM [Fe(CN)_6_]^3−/4−^ diluted in 0.01 M PBS (pH 7.4) at the room temperature.

### 2.2. Preparation of the Genosensor

All SPGE were electrochemically cleaned in the presence of a 10 mM HCIO_4_ pH 4 ± 0.2. The electrochemical cleaning method used was cyclic voltammetry with a potential range of −400 mV to +1000 mV, 4 cycles and a scanning rate of 100 mV/second.

To produce a functional layer on Au electrode surface, the working electrode was incubated for 30 min, in 40 *μ*L of 100 *μ*M thiolated probe sequence (ssDNA) and a thiolated compound (we tested three compounds: 2-mercaptoethanol, 6-mercapto-1-hexanol and 11-mercaptoundecanoic acid) 1 *μ*M (3 : 1) diluted in PBS 0.01 M. After this step, the SPGE was rinsed with 0.1% SDS diluted in PBS, and then with Milli-Q ultrapure water to remove the nonadsorbed residuals. In this way, the working gold electrodes become modified with MUCS, MERS, and MCHS adlayer (MER + ssDNA = MERS, MCH + ssDNA = MCHS and MUA + ssDNA = MUCS).

### 2.3. Hybridization Assay

This study used the method of Garcia-Melo et al. and the method's description partly reproduces their wording [31]. After choosing the best thiol (MUCS), we used it to work in all processes. The resulting electrodes were incubated in the desired concentrations of fully complementary target (K-ras) solutions for 30 min to allow probe-DNA hybridization, followed by rinsing with 0.1% SDS diluted in PBS, as well as with Milli-Q ultrapure water to remove the unbound targets. Subsequently, the electrodes were subjected to electrochemical measurements. The Au/MUCS/dsDNA modified electrode surface was incubated in 10 *μ*L of 20 *μ*M (diluted in PBS) Dox hybridization indicator for 20 min at room temperature in the dark, followed by rinsing three times, first with Milli-Q ultrapure water and then with 0.01 M PBS. Subsequently, the electrodes were subjected to electrochemical measurements.

### 2.4. Hybridization Temperature

The efficiency of the hybridization was evaluated to maximize the sensitivity of genosensor. The SPGE previously modified (Au/MUCS) was hybridized with the fully complementary target at different temperatures such 20, 37, 40, 45, 50, and 55°C, using the US autoflow automatic CO_2_ (NU-4750) incubator (NuAire, Plymouth, MN). After the hybridization reaction, each electrode was incubated in 10 *μ*L of 20 *μ*M Dox (diluted in PBS), hybridization indicator for 20 min at the room temperature in dark, followed by rinsing three times, first with Milli-Q ultrapure water and then with 0.01 M PBS. Subsequently, the electrodes were subjected to electrochemical measurements. It is in accordance with the method previously described by Garcia-Melo et al. [31].

### 2.5. Selectivity of the Genosensor

Selectivity is a crucial factor to evaluate the performance of a genosensor. The device previously modified (Au/MUCS) was incubated with non-complementary sequences (sNC) solution. Later, the genosensor was incubated with a fully complementary target (K-ras). Moreover, previously modified electrodes (Au/MUCS/sNC and Au/MUCS/dsDNA) were incubated with 10 *μ*L of 20 *μ*M Dox solution in PBS 0.01 M during 20 min at room temperature. Subsequently, the electrodes were subjected to electrochemical measurements. Details of the selectivity evaluation were previously described by Garcia-Melo et al. [31].

### 2.6. Electrochemical Analysis

All electrochemical measurements were made by means of Cyclic Voltammetry (CV) at room temperature with a Bioanalytical Systems BAS-100 electrochemistry workstation (West Lafayette, IN) using Screen-Printed Gold Electrodes (SPGE) (DropSens: DRP 250AT; (Oviedo, Spain) with conventional three-electrode system consisting of an Au as the working electrode (4 mm diameter) and the geometric area is 0.126 cm^2^, Pt as the counter electrode, and Ag/AgCl as the reference electrode. CV analyses were performed in the redox probe solution by scanning the potentials from −400 mV to +500 mV, at a scan rate of 50 mVs^−1^. A new SPGE was used for each assay and each assay was done five times.

### 2.7. Atomic Force Microscopy Analysis

Studies of surface morphology of the SPGE gold electrode were performed with Atomic Force Microscopy (AFM, Nanoscope III Multimode SPM, Digital Instruments, Santa Barbara, California), in the tapping mode.

The surfaces of modified and unmodified SPGE were characterized at room temperature using the tapping mode and standard silicon probes with the following features: resonance frequency (fo) of 61–85 kHz, spring constant (*k*) of 0.5–4.4 N/nm, with scan rate of 0.5 Hz. The acquisition modes of the AFM images were height and phase. Area of 1 *μ*m × 1 *μ*m with *Z* scale 0–250 nm was used to calculate the Root Mean Square roughness (RMS_Rq_) to find the morphological surface changes in function of the modified process.

## 3. Results and Discussion

**Figure 1:**
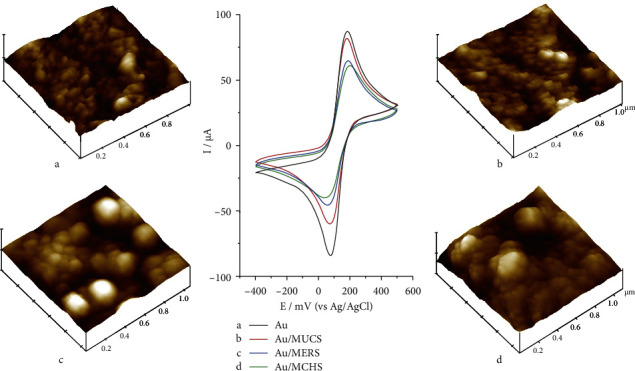
Cyclic voltammograms current of the 20 mM [Fe(CN)_6_]^−3/−4^ redox probe/0.01 M PBS (pH 7.4) and AFM image of 3D morphology of (for both cases) registered on unmodified Au electrode (a), modified Au/MUCS electrode (b) modified Au/MERS electrode and modified Au/MCHS electrode, with area of 1 *μ*m × 1 *μ*m and *Z* scale 0–250 nm.

Figure 1 shows the CV obtained by the [Fe(CN)_6_]^−3/−4^ probe on SPGE unmodified and modified with alkylthiols (MUCS, MERS, and MCHS), with images obtained with AFM on SPGE for each of the modifications (MUCS, MERS, MCHS). Regarding CV, the maximum current of [Fe(CN)_6_]^3−/4−^ of the anodic peak was obtained with SPGE without modification (Au) at 86.79 *μ*A, (line a). On the other hand, to evaluate the surface of SPGE with AFM, it was determined the root mean square roughness (RMS_[Rq]_) that describes the morphological properties and altitude of the topographic composition of the unmodified SPGE, and the result obtained was of 9.38 nm of RMS_[Rq]_, Figure 1(a). In addition, regions were found with particle sizes of 28 nm; this indicates the unmodified SPGE is not uniform and contains a high roughness, and granular surface, possibly produced by the process of impression, as showed by Hempen et al. [32], where they carried out an analysis with AFM and found very similar results to those obtained in this study. Similarly, Jalalian et al. [33] verified the morphology of the surface of Au, and these results coincide with previously obtained by this research. It has been observed that a surface containing smaller grains of gold facilitates the adsorption of a greater amount of molecules that contain alkylthiols [34]. With respect to the modifications carried out with the alkylthiols, ssDNA, and MUCS (Figure 1, line b), these presented a favorable electrochemical response for the construction of this type of genosensor, due to that the maximum anodic peak decreased of 6.16% *I*_Relative_. In contrast, MERS and MCHS showed a decrease of 25.74 and 29.96% *I*_Relative_, respectively (Figure 1, lines c and d). The results show that the current decrease was due to the blockage of electron flow produced on the electrode surface by ssDNA and alkylthiols. The above could be explained as follows: (1) spatial inhibition-physical obstruction of ions to reach the electrode surface and (2) electrostatic inhibition of the ion transfer by the increase of negative charge on the electrode surface [35]. The previous effect was at a different proportion for each of the alkylthiols; it depends on the size of the chain, on the terminal group, and on the density of the packaging. On another hand, Raymundo-Pereira et al. [36] observed that the SAMs formed from ethanolic solution or from aqueous solutions at pH 12.5 are ideal conditions for nanostructuring in the development of genosensors. In this case, MUCS was able to detect the target DNA structure under experimental conditions. Nevertheless, the other alkylthiols that were prepared in aqueous solutions at pH 7.4 showed a change of current but not significant, probably by non-specific interactions produced due to the protonation of SAMs and an unfavorable chemical environment by pH solution [36].

In this respect, Miranda-Castro et al. [37] mentioned that the utilization of short-chain alkylthiols presents some limitations ranging from restriction of the space between the ssDNA and the alkylthiols (steric effect), as well as the electrostatic repulsion produced by the terminal groups OH that provide more defective and less ordered SAM. Likewise, it has been observed that employing MCH at a pH of 7.4 (physiological) could be capable of providing a high negative charge and avoiding the adsorption of the ssDNA on the electrode surface [32, 37]. In addition, two problems were observed: (i) the strands of the probes are very tight, and these, in some cases, do not carry out the hybridization reaction as a consequence of restriction of space (steric effects) and electrostatic repulsions of the strands (charge effects), and (ii) few single-stranded ssDNA probes bound to the surface afford little accessibility to the target strands or to the complementary strands (dsDNA), thus producing little or null detection [38]. In this context, Cecchet et al. [26] observed that short-chain SAM present some inconveniences about their long-chain counterparts due to the following: (i) a higher double-layer capacitance that makes the signal-to-background ratio less favorable; (ii) a lower discrimination ability toward potential interferents, and (iii) less reproducibility and robustness of the devices. Moreover, the high hydrophobic interactions lead to greater monolayer coverage, and the heterogeneous hydrophobic and hydrophilic interactions lead to poor surface coverage on the surface [39]. On analyzing and comparing the MUCS modifications, it was observed that these are more effective for our process, because it allowed to block the flow of current and obtain a desired electrochemical response, suggesting that the surface densities are ideal for our schema of immobilization and formation of genosensors. That could have been due to COOH terminal groups, and the steric impediment produced, it allowed for the perpendicular packaging of the ssDNA, adopting a parallel position for these to extend into the solution or onto the surface of the electrode, remaining exposed and ready to carry out detection and/or the hybridization reaction [40, 41]. Regarding hydrophobic reactions, Monserud and Schwartz [42] reported that the longitude for this type of compound (MUCS) permits the adoption of conformations that minimize the exposed area available for interacting with the surface, thus allowing the chains to interact in the hybridization process. In addition, the hydrophobic interactions that exist between MUC and ssDNA produce a denser SAM, at the same time increasing the number of immobilized ssDNA on the electrode surface with greater flexibility, necessary for effective hybridization [43]. In other words, the further away this immobilized molecule is from the surface, the nearer it is to the solution; therefore, it is more probable that it would react freely with the molecules [44]. Analyzing AFM images from SPGE previously modified, the results demonstrated that the surface with less roughness was MUCS (9.00 nm RMS_[Rq]_) (Figure 1(b)); furthermore, particle sizes were found ranging from 27 to 52 nm. These data suggest that the oligonucleotides extend to a greater degree in a more steric hindrance area, as indicated by Satjapipat et al. [34], where the researchers were able to observe that steric congestion in a highly localized area reduces variation in the transversal sections of the DNA chains. MERS (Figure 1(c)), the RMS_[Rq]_ was 12.78 nm with particulate sizes of 249, 48, and 13 nm. And MCHS presented an RMS_[Rq]_ of 17.32 nm with particulate sizes of 42 and 69 nm (Figure 1(d)). These results suggest that there could be a correlation between the current and the roughness of the electrode surface. In other words, the higher the roughness, the lower the electron flow or the higher the current blockage, in this case only.

**Figure 2:**
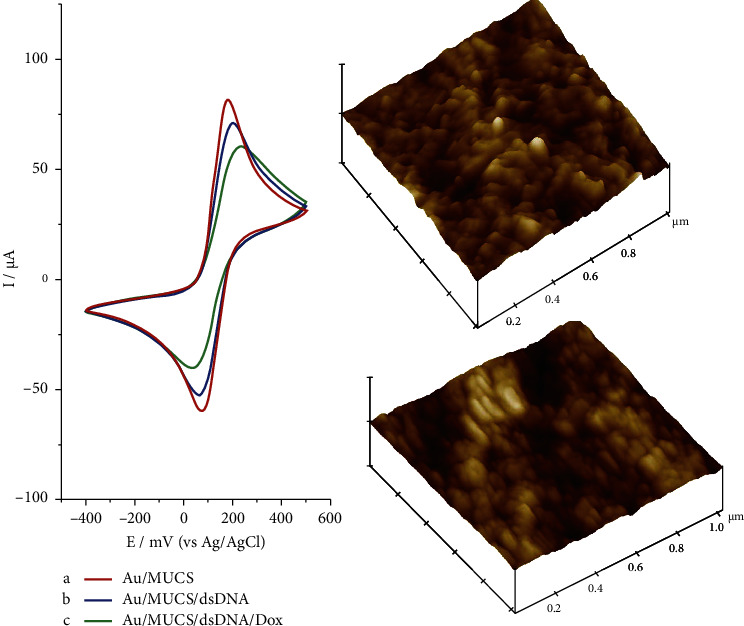
Cyclic voltammograms current of the 20 mM [Fe(CN)_6_]^−3/−4^ redox probe/0.01 M PBS (pH 7.4) registered on modified Au/MUCS (a), 10^−6^ M Au/MUCS/dsDNA electrode (b), and Au/MUCS/dsDNA/Dox electrode (c). AFM image of 3D morphology of registered on Au/MERS/dsDNA electrode (b) and modified Au/MCHS/dsDNA/Dox electrode (c), with area of 1 *μ*m × 1 *μ*m and *Z* scale 0–250 nm.

Based on the results obtained with MUCS and the data reported in the Garcia-Melo et al. [31] research, the hybridization reaction was evaluated.  Figure 2 depicts the CV obtained by the [Fe(CN)_6_]^−3/−4^ probe on SPGE previously modified with MUCS, the hybridization reaction and the interaction of Dox with double-stranded DNA. Additionally, the images obtained with AFM on the SPGE from these two latter processes are shown (dsDNA and Dox). In the case of the CV (Figure 2, line a), the maximum current peak of [Fe(CN)_6_]^−3/−4^ was obtained from SPGE modified with Au/MUCS and it presented a maximum anodic peak of 81.44 *μ*A. It is noteworthy that, during this process and in later assays, the values obtained for Au/MUCS represent 0% of hybridization reaction with the DNA target (*K-ras* gene). Thus, any diminution of the maximum anodic peak of MUCS is indicative of the hybridization reaction, as well as the increase in resistance to interfacial ion transfer [Fe(CN)_6_]^−3/−4^ [31]. In the hybridization reaction detection process, a significant decrease of 16.17% *I*_Relative_ for Au/MUCS/dsDNA with respect to Au/MUCS was observed. This effect can be related to then following two factors: (1) restriction of the diffusion of [Fe(CN)_6_]^−3/−4^ directly on the gold's surface, and/or (2) after hybridization, the Faradaic current for [Fe(CN)_6_]^−3/−4^ decreased even more due to the density increased negative charge, and to the molecular overcrowding of the electrode‒film interface [37, 45, 46]. Carr et al. [47] observed that the redox probes ([Fe(CN)_6_]^−3/−4^) did not reach the surface electrode due to electrostatic repulsion, the effect was similar for this investigation. Another factor that possibly exerted an influence was the formation of hydrogen bonds between the complementary sequences, in which it is more difficult to oxidize the bases, and consequently to decrease the maximum current after hybridization [48]. These observations coincide with those previously reported in the literature [31, 35, 46, 49, 50]. After hybridization reaction, it was evaluated the capacity of Dox (intercalating agent and hybridization indicator) on the previously modified SPGE. In Figure 2, line c, it was observed that the maximum anodic peak decreased 37.02% *I*_Relative_ with respect to Au/MUCS. These results confirm the capacity of Dox for indicating the hybridization process, and these coincide with other studies in which the researchers mentioned that the response can be related with the capacity of Dox to interact with double-stranded genetic material and, at the same time, to stabilize dsDNA structures. Furthermore, a complex could be formed with the phosphate groups, thus increasing the electrostatic charge, in turn blocking the transfer of ions toward the surface of the electrode, which led to a current decrease, as shown in Figure 2, line c [31, 51–56]. Regarding the analysis conducted by AFM, it has been demonstrated that this is a technique that provides images with high resolution and extraordinary precision, capable of analyzing molecules of DNA and SAM, as well as those obtained in this investigation [57].

Using the AFM images, we evaluated changes in the topography and calculated the roughness _[Rq]_ of each electrode surface. In Figure 2(b), it is observed that the image is obtained from the modified SPGE (Au/MUCS/dsDNA) after the hybridization reaction, whose RMS_[Rq]_ is 10.19 nm. On comparing this result with Au/MUCS, the increase of the RMS_[Rq]_ is observed, as well as an increase in particle number and size, which range from 79 to 124 nm. On the other hand, after the interaction of Dox with the double strand of DNA, RMS_[Rq]_ decreased to 9.56 nm and only particles of 81 nm were observed. It is well known that variations in surface roughness values can be explained by the inclusion of modifying materials on the surface [58], that is, these changes are due to the presence of double-stranded oligonucleotides, which are wider and less flexible than ssDNA and are consequently capable of forming larger structures [35]. Another factor that we must consider is the coating previously performed with MUC and ssDNA on the surface of the SPGE because all of this taken together produces different particulate sizes with larger groups and is reflected in a less homogeneous surface [48]. These results suggest that the hybridization reaction process was carried out, which is confirmed by the data obtained in the electrochemical study. Within this context, Kaushal et al. [59] observed similar results to those obtained in this investigation. The researchers observed that the change in the surface morphology and the increase in roughness of the gold working electrode, it was parameters that confirmed the immobilization of the thiolated DNA probes and the hybridization reactions. The same occurred in this research, where the RMS_[Rq]_ increased gradually according to the stage in which the working electrode was modified [59, 60]. Dash et al. [61, 62] confirmed the latter asseveration, suggesting that the change in morphology and the increase in roughness establish that the immobilization of the probe and of the hybridization reaction have been carried out on the electrode [60–62]. In regards to Dox, these results suggest that, on interacting with the double-chain DNA, a strong complex is formed between these molecules. Some authors mention that Dox linked between adenine and guanine in position N7 through the formation of hydrogen bonds between the purine and the Dox hydroxyl groups [63]; it is possible that, as a consequence of the latter, a diminution is produced in roughness, as well as an increase in the packaging of the genetic material, and changes in the morphology are presented. In other words, when Dox is intercalated in the DNA, a lengthening is produced of the dsDNA, which impedes ion transfer to the electrode's surface, giving rise to an increase in the steric impediment, which is only produced when the hybridization reaction has been carried out [64]. Therefore, the layer of the electrode's surface increases and, at the same time, Dox allows us to increase the sensitivity of the device. In addition, it allows us to confirm that the hybridization reaction was produced adequately on the surface of the electrode, as observed in the previously reported work [31].

**Figure 3:**
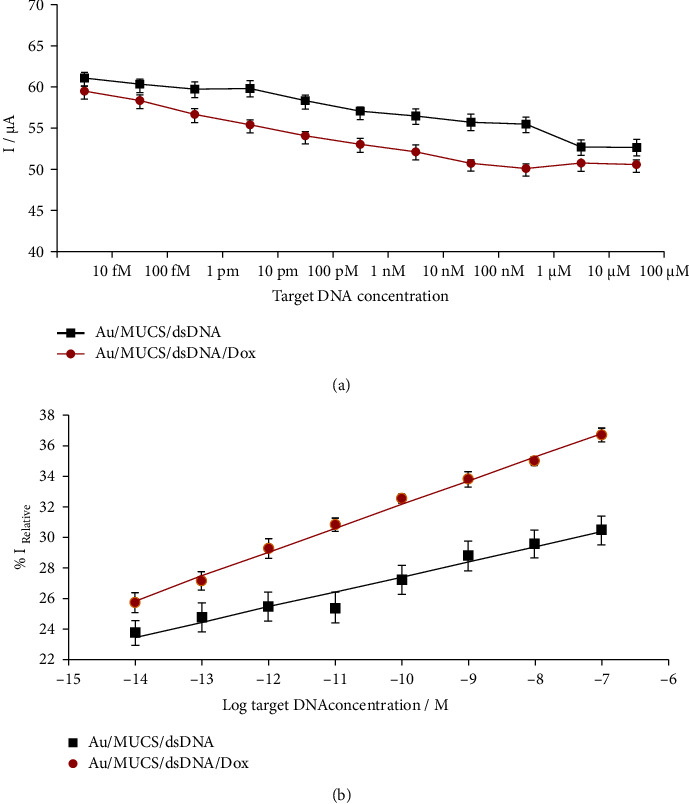
(a) Anodic peak current for [Fe(CN)_6_]^3−/4−^ redox probe recorded at Au/MUCS/dsDNA and Au/MUCS/dsDNA/dox electrodes, in different concentration of target DNA, respectively. (b) Linear relationship between the relative current change of the anodic peak [Fe(CN)_6_]^3−/4−^ redox probe and the logarithm of the target DNA concentration at Au/MUCS/dsDNA and Au/MUCS/dsDNA/Dox electrodes, respectively.

The analytical performance of the genosensor was evaluated using different concentrations of target DNA from 10 fM to 100 *μ*M.  Figure 3(a) (black line) represents the maximum current anodic peaks of [Fe(CN)_6_]^−3/−4^ obtained from the cyclic voltammograms of each dsDNA target concentration used in the hybridization process. In the black line (Figure 3(a)), the maximum current value for Au/MUCS/dsDNA was found at 61.13 *μ*A and the minimum current value was at 52.65 *μ*A due to an increase in the concentration of target DNA on the electrode.  Figure 3(a) (red line) represents the analytical behavior of the nanosensor when adding Dox at 20 *μ*M (diluted in PBS) for 20 min at room temperature after the hybridization reaction for each concentration of the dsDNA target. The current decreased from 59.55 *μ*A to 50.65 *μ*A (Au/MUCS/dsDNA/Dox) and this result establishes and confirms that the use of Dox improved the sensitivity of our genosensor, as shown in the previous study [31].

To determine the capacity of the genosensor, we calculated the relative proportion of the decrease of the anodic peak versus the concentration of the dsDNA target, within the range of 10 fM to 1 *μ*M. For each concentration, the hybridization capacity was calculated with the following equation: % *I*_Relative_ = [(*I*_0_ − *I*_*m*_)/*I*_0_]*∗*100, where *I*_0_ is the maximum anodic peak of Au/MUCS, and *I*_*m*_ corresponds to the maximum anodic peak of current after the hybridization reaction (Au/MUCS/dsDNA).  Figure 3(b) presents the linear correlation between the logarithms for each concentration of the dsDNA target (Au/MUCS/dsDNA) and the *I*_Relative_ (%), whose linear regression coefficient is *R*^2^ = 0.9862 (black line). A gradual decrease in the current percentage was observed with respect to the increase of the DNA target concentration. The detection limit for Au/MUCS/dsDNA was determined according to the 3 × Sb criterion [31], where Sb was estimated as the Standard deviation (SD) of the blank measurements, and it was adjusted to the respective equation of the linear portion of the graph. The detection limit was measured at 3.36 × 10^−14^ M and the regression equation was the following: % *I*_Relative_ (*μ*A) = 37.26 + 0.98 log[DNA target] (M). We compared this work with other biosensors for *K-ras* gene detection, and this biosensor exhibits a very wide linear range and a lower detection limit than other devices previously reported, in addition to being simpler, faster, and easier to develop [65–71]. In this sense, the researchers have observed that the construction of genosensors developed with MUA-based matrixes are capable of detecting target DNA in the concentration range between 1.0 × 10^−18^ and 1.0 × 10^−6^ M [72], as built in this project and the results show that the genosensor could be used for DNA detection with good sensitivity.

In relation to Dox, we followed the procedure previously described, and we determined the linear correlation between the logarithm of the dsDNA concentration after Dox interaction versus the *I*_Relative_ (%). Whose linear regression coefficient corresponded to 0.9975 (Figure 3(b), red line). The detection limit corresponded to 7.96 × 10^−15^ M (S/N = 3), and the regression equation was the following: % *I*_Relative_ (*μ*A) = 47.80 + 1.56 log[DNA target] (M).

These results showed an adequate sensitivity to identify the target *K-ras* mutation; however, when Dox was utilized as an indicator of the hybridization reaction, we observed an increase in the capacity of detection, reaching lower concentrations of dsDNA. Thus, the use of Dox improves the sensitivity of the genosensor. Furthermore, the gradual decrease of the electrochemical response is associated with a reduction in the flow velocity of electrons through the biorecognition layer, factors that increase with the use of Dox [73, 74].

**Figure 4:**
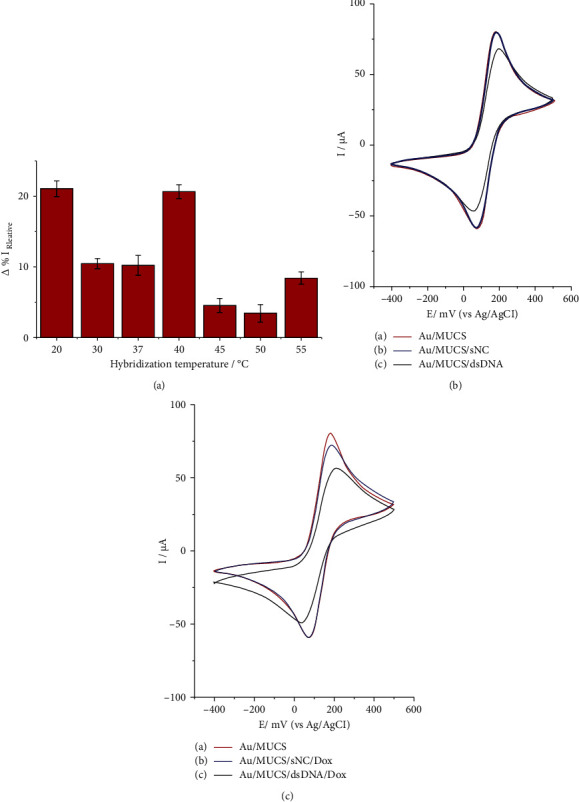
(a) The relative current change of the anodic peak of [Fe(CN)_6_]^−3/−4^ redox probe versus hybridization temperature after the dox treatment, at the room temperature (20°C) (Au/MUCS/dsDNA/dox). (b) Cyclic voltammograms of [Fe(CN)_6_]^3−/4−^ redox probe recorded at Au/MUCS electrode (a), Au/MUCS/sNC electrode (b), and Au/MUCS/dsDNA electrode (c). (c) Cyclic voltammograms of [Fe(CN)_6_]^3−/4−^ redox probe recorded at Au/MUCS electrode (a) Au/MUCS/sNC/dox electrode (b), and Au/MUCS/dsDNA/dox electrode. The dox solution was added in excess 10^−5^ (M) all electrochemical measures were made by means of cyclic voltammetry (CV) at a scan rate of 50 mV/s, within a potential range from −400 mV to +500 mV.

To obtain a better analytical performance, we investigated different hybridization temperatures of the target DNA and evaluated the interaction of Dox as an indicator of the hybridization reaction.  Figure 4 presents the effect of Dox on the hybridization temperature in the electrochemical response of the genosensor. The bars represent the maximum anodic peak (% *I*_Relative_) obtained by the [Fe(CN)_6_]^−3/−4^ probe on SPGE modified with Au/MUCS/dsDNA/Dox, after the hybridization process at different temperatures. It is noteworthy that we added Dox at 20 *µ*M (diluted in PBS solution) during 20 min at room temperature in the dark, over the previously hybridized electrodes (Au/MUCS/dsDNA). The maximum difference in current was 21.06 and 20.62% *I*_Relative_ at temperatures of 20 and 40°C, respectively. The best hybridization of dsDNA has been shown to occur at temperatures close to the melting point, it happened with the temperature at 40°C. However, when we employed Dox, the sensitivity of the sensor improved; it is possible that a great number of Dox molecules were intercalated after each target DNA was hybridized, accumulating on the dsDNA at the temperature of 20°C [75, 76]. In view of these results, we decided to employ the 20°C temperature for later assays.

Figure 4(b) shows the cyclic voltammograms of SPGE modified with Au/MUCS, Au/MUCS/sNC, and Au/MUCS/dsDNA. Figure 4(b), line a indicates the maximum anodic peak of [Fe(CN)_6_]^−3/−4^ after modification with Au/MUCS, and this demonstrated values of 80.16 *μ*A at a potential of 182 mV. This result is in agreement with the data previously obtained of Au/MUCS. Figure 4(b), line b represents the interaction of the genosensor with Au/MUCS/sNC (the Non-Complementary DNA sequence). For these results, we obtained a diminution of only 1.0% *I*_Relative_; the current decrease indicates that the hybridization reaction on the sNC is not being carried out. Contrariwise, Figure 4(b) line c shows the hybridization reaction of Au/MUCS/dsDNA with the diminution of the maximum anodic peak up to 16.94% *I*_Relative_ with respect to Au/MUCS, and 15.94% *I*_Relative_ with respect to Au/MUCS/sNC. It is the same as with earlier obtained data and establishes that the genosensor possesses the faculty of discerning between dsDNA and sNC. Therefore, the device exhibits excellent selectivity in terms of another type of sample from those evaluated.

Figure 4(c) presents the cyclic voltammograms of SPGE modified with Au/MUCS (Figure 4(c), line a), while lines b and c indicate the same process in SPGE modified with Au/MUCS/sNC and with Au/MUCS/dsDNA after the interaction with Dox. In Figure 4(c), line b (Au/MUCS/sNC/Dox) reveals a maximum anodic peak with a decrease 10.08% *I*_Relative_ with respect to Au/MUCS. Contrariwise, when Dox was added to Au/MUCS/dsDNA/Dox, the maximum anodic peak (Figure 4(c), line c) decreased 33.98% *I*_Relative_ with respect to Au/MUCS and 23.90% *I*_Relative_ with respect to Au/MUCS/sNC/Dox. These results confirm that Dox is indeed carrying out its function as indicator of the hybridization reaction, intercalating with the double chains of DNA and blocking the flow of the ions of [Fe(CN)_6_]^−3/−4^. It is important to mention that Dox is a useful tool to increase the selectivity of this type of electrochemical genosensors. Thus, it could be concluded that our device is highly selective for detecting the *K-ras* gene. In future investigations, it is necessary to carry out more analysis with the purpose of improving the analytical potential of this genosensor.

Seven genosensor were fabricated in parallel, under the same conditions for the reproducibility of electrochemical genosensor by using 1 × 10^−11^ M target sequence in solution. The Relative Standard Deviation (RSD) was 3.7%, indicating a satisfactory reproducibility of the fabricated electrochemical genosensor. The stability of the electrochemical genosensor was investigated by storing it at 4°C and measured every 7 days. After 3 weeks, it retained 94% of its initial current response, indicating that the construction of electrochemical genosensor was stable.

## 4. Conclusions

The nanostructured electrochemical genosensor was built based on modification of the SPGE surface with MUCS for the detection of the k12p.1 mutation of the K-ras gene. We identified the complementary sequences of this gene, and quantified these with a high degree of selectivity and sensitivity. This device is capable of detecting, in solution, this gene within concentrations ranging from 10 fM to 1 *µ*M. The procedure improved its sensitivity and selectivity after adding Dox (indicator and enhancer of the DNA hybridization process) presenting a detection limit of 7.96 fM. In addition, this molecule allowed us to carry out the hybridization reaction at room temperature. This genosensor possesses simple, accessible, and stable fabrication with a rapid response and with satisfactory reproducibility. The results presented suggest that our methodology would be adequate for the detection of the k12p.1 mutation of the *K-ras* gene in real samples.

## Data Availability

The data that support the findings of this study are available from the corresponding author upon reasonable request.
